# Supplementary material for: A Novel Self-Assembled Graphene-Based Flame Retardant: Synthesis and Flame Retardant Performance in PLA
Source: Polymers (Basel). 2021 Dec 1;13(23):4216. doi: 10.3390/polym13234216 (PMC8659536; doi:10.3390/polym13234216)
Supplement: Supplementary file 1 [file polymers-13-04216-s001.zip › polymers-1478106-supplementary.pdf]

Supplementary Materials

A novel self-assembled graphene-based flame retardant: synthesis and flame retardant performance in PLA

Peixin Yang , Hanguang Wu,Feifei, Yang, Jie Yang, Rui Wang and Zhiguo Zhu**

((Optional Dedication))
P. X. Yang, Dr H. G. Wu, F. F. Yang, X. J. Yang, Prof. R. Wang, Prof. Z. G. Zhu

School Materials Design and Engineering, Beijing Institute of Fashion Technology, 100029, Beijing, China
Beijing Key Laboratory of Clothing Materials R & D and Assessment, Beijing Institute of Fashion Technology, 100029, Beijing, China

Table of contents

1. Supplementary Tables
2. Supplementary Figures

Supplementary Tables

Table S1 Compositions of flame-retarded PMrG

Sample	rGO (g)	Melamine (g)	PA (g)
PMrG-1	1	0.5	2.5
PMrG-2	1	0.8	4
PMrG-3	1	1	5
PMrG-4	1	2	10
PMrG-5	1	5	25

Table S2 TGA data of rGO, PM, and PMrG-3

Sample	$T_{5\%}$ (°C)	T_{max} (°C)	RW (wt%)
rGO	243.0	433.8	38.4
PM	209.9	255.8	38.1
PMrG-3	214.3	384.1	45.0

Table S3 TGA data of PLA and its composites.

Sample	$T_{5\%}$ (°C)	T_{max} (°C)	RW (%)
PLA	321.1	357.6	0
rGO/PLA	338.6	367.5	7.8
PM/PLA	319.1	368.4	4.2
PMrG-1/PLA	327.3	370.3	2.7
PMrG-2/PLA	325.1	364.6	4.1
PMrG-3/PLA	323.5	370.1	6.3
PMrG-4/PLA	327.4	369.4	5.5
PMrG-5/PLA	330.1	368.9	1.9

Table S4 LOI and UL-94 test results of the pure PLA and the PMrG/PLA composites with different PMrG composition

Sample	LOI (%)	UL-94 rating
PLA	19	No rating
rGO/PLA	22	V-1
PM/PLA	23	No rating
PMrG-1/PLA	23	V-2
PMrG-2/PLA	24	V-1
PMrG-3/PLA	25	V-0
PMrG-4/PLA	25	V-0
PMrG-5/PLA	24	V-1

Table S5 Cone calorimeter data of PMrG/PLA composites

Sample	TTI(s)	pHRR(Kw/m^2)	THR(MJ/m^2)	TSR(m^2/m^2)	Char residue (%)
PMrG-1/PLA	46	317.8	53.3	400.3	6.0
PMrG-2/PLA	51	283.5	52.1	310.1	9.2
PMrG-3/PLA	59	276.1	46.5	191.5	15.3
PMrG-4/PLA	55	277.6	47.4	262.6	9.3
PMrG-5/PLA	52	298.8	53.4	383.7	6.6

2. Supplementary Figures

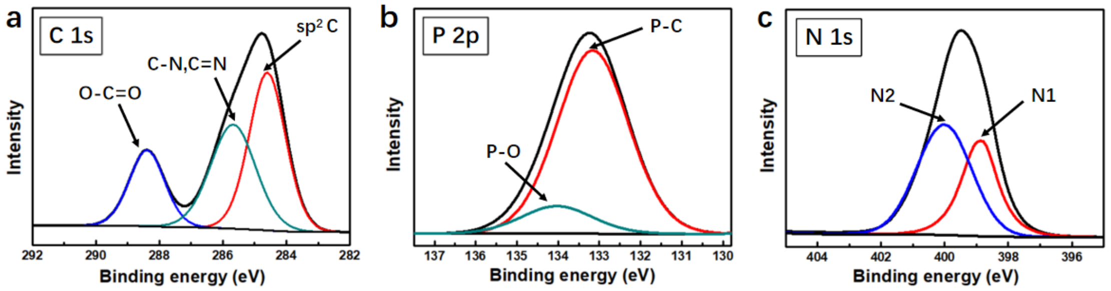

Figure S1 High-resolution XPS spectra for (a) C 1s, (b) N 1s, and (c) P 2p.

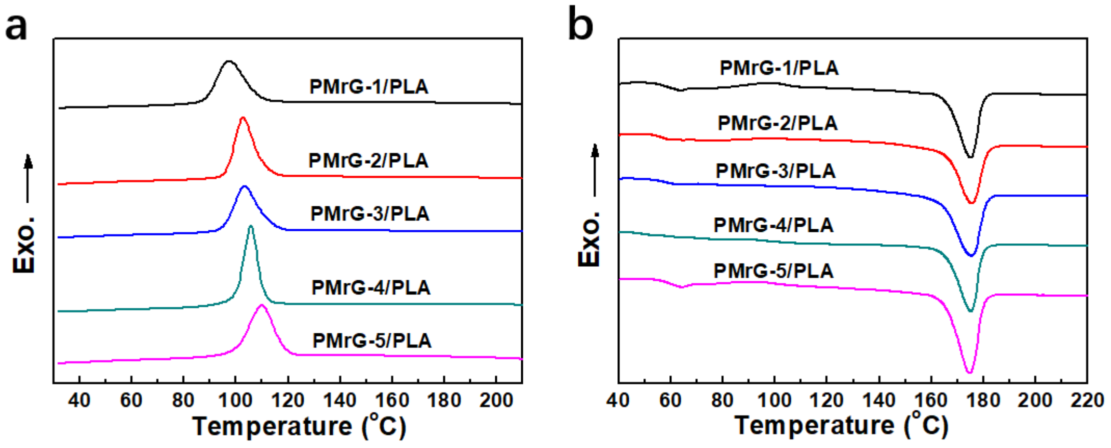

Figure S2 DSC curves of PMrG/PLA compositions with different compositions during the (a) cooling and (b) heating process (the mass fractions of the additives in the composites are all 10%).

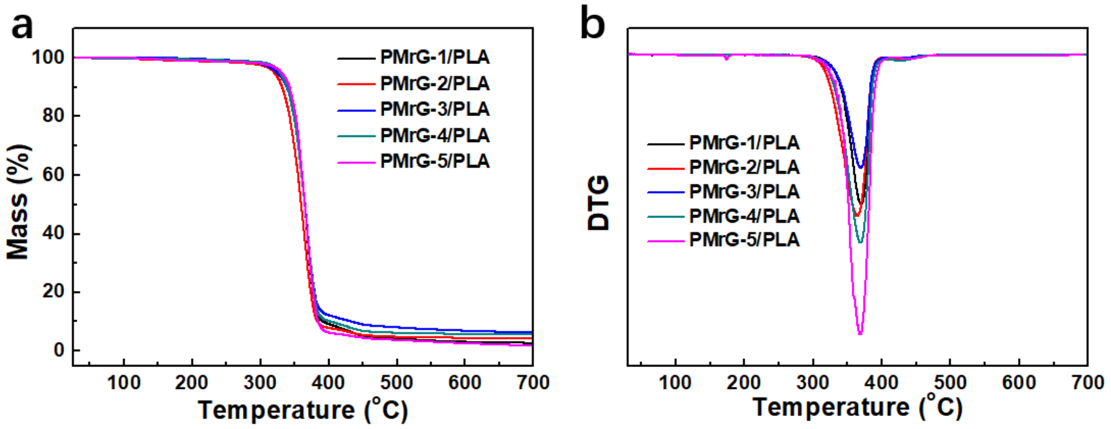

Figure S3 (a) TGA and (b) DTG curves of the PMrG/PLA composites with different PMrG compositions (the mass fractions of the additives in the composites are all 10%).

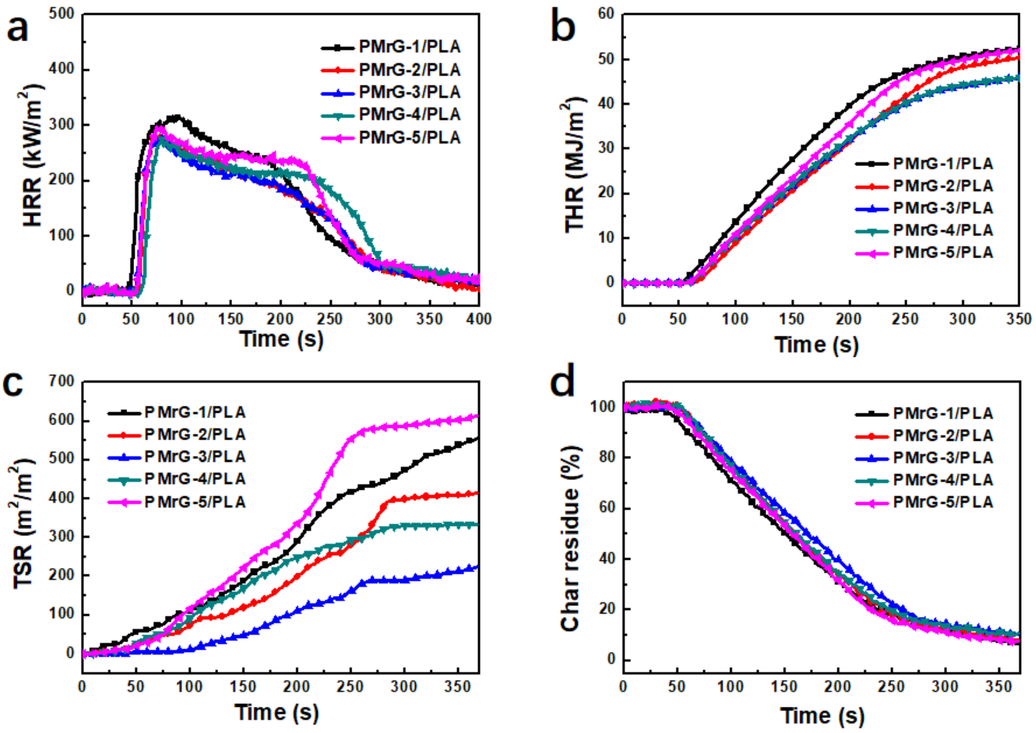

Figure S4 (a) Heat release rate, (b) Total heat release, (c) Total smoke release, and (d) Char residue curves of PMrG/PLA composites with different PMrG compositions (the mass fractions of PMrG in the composites are all 10%).
